# Review of Urate-Lowering Therapeutics: From the Past to the Future

**DOI:** 10.3389/fphar.2022.925219

**Published:** 2022-08-23

**Authors:** Christopher Jenkins, Jennifer H. Hwang, Jeffrey B. Kopp, Cheryl A. Winkler, Sung Kweon Cho

**Affiliations:** ^1^ Department of Internal Medicine, University of Connecticut Health Center, Farmington, CT, United States; ^2^ Department of Internal Medicine, The Hospital of Central Connecticut, New Britain, CT, United States; ^3^ Kidney Disease Section, National Institute of Diabetes and Digestive and Kidney Diseases, National Institutes of Health, Bethesda, MD, United States; ^4^ Basic Research Laboratory, Center for Cancer Research, National Cancer Institute, Leidos Biomedical Research, Frederick National Laboratory, Frederick, MD, United States; ^5^ Department of Pharmacology, Ajou University School of Medicine, Suwon, South Korea

**Keywords:** uric acid, ULT, review, drug, gout

## Abstract

We reviewed all currently available ULT, as well as any medications in development using following databases: United States Food and Drug Administration (FDA), European Medicines Agency (EMA), Japanese Pharmaceutical and Medical Devices Agency (PMDA), and ClinicalTrials.gov. We identified a total of 36 drugs, including 10 approved drugs, 17 in clinical testing phases, and 9 in preclinical developmental phases. The 26 drugs currently undergoing testing and development include 5 xanthine oxidase inhibitors, 14 uricosurics, 6 recombinant uricases, and one with multiple urate-lowering mechanisms of action. Herein, we reviewed the benefit and risk of each drug summarizing currently available drugs. New trials of uricosuric agents are underway to develop the new indication. New drugs are going on to improve the potency of recombinant uricase and to develop the new route administration of such as oral formulation. This review will provide valuable information on the properties, indications, and limitations of ULTs.

## Introduction

Gout is one of the most common forms of inflammatory arthritis (Chen-Xu et al., 2019). Gout is typically characterized by hyperuricemia, with a serum uric acid (SUA) greater than 6.8 mg/dl. Hyperuricemia leads to the formation of urate crystals within the joint space, which triggers an immune response mediated by IL-1β *via* the NOD-like receptor pyrin domain-containing protein 3 (NLRP3) inflammasome. Hyperuricemia occurs as a result of overproduction or underexcretion of urate, with 90% of cases classically attributed to the latter. In humans, two-thirds of urate is excreted in the urine and one-third is excreted through the gastrointestinal tract; the fraction cleared by the GI tract increases in chronic kidney disease (CKD) (Sorensen, 1965; Bhatnagar et al., 2016; Nigam and Bhatnagar, 2018). Hyperuricemia is associated with several chronic conditions including CKD, hypertension, cardiovascular disease (CVD), stroke, diabetes, and metabolic syndrome (Culleton et al., 1999; Choi et al., 2005; Coutinho et al., 2007; Storhaug et al., 2013; Zalawadiya et al., 2015; Xu et al., 2016). Increased SUA have shown to associate with CVD, however the causal relationship between increased SUA and CVD remains controversial (Coutinho et al., 2007; Saito et al., 2021).

Currently, there are only five FDA-approved and manufactured urate-lowering therapeutics (ULTs): allopurinol, febuxostat, probenecid, rasburicase, and pegloticase. Each of these medications has limitations that prevent widespread use: allopurinol can cause severe allergic reactions, febuxostat has a black-box warning of increased risk of cardiovascular death, probenecid can precipitate nephrolithiasis and has several drug-drug interactions, rasburicase is only approved for hyperuricemia in malignancy, and pegloticase is costly and has a black-box warning for anaphylaxis. Lesinurad and sulfinpyrazone are FDA-approved ULTs, but are not longer commercially available. Therefore, there is an urgent need for novel ULTs.

In this narrative review, we will discuss all medications currently available for the treatment of hyperuricemia and gout, and introduce novel ULTs that are currently in development.

## Methods

We reviewed all approved ULTs and organized them according to the mechanism of action (Table 1). We searched the approved drug databases maintained by the US Food and Drug Administration (FDA) (https://www.accessdata.fda.gov/scripts/cder/daf/index.cfm), European Medicines Agency (EMA) (https://www.ema.europa.eu/en/human-regulatory/post-authorisation/data-medicines-iso-idmp-standards/public-data-article-57-database), and Japanese Pharmaceutical and Medical Devices Agency (PMDA) (https://www.pmda.go.jp/english/index.html). Based on the results of these searches, we selected ten medications to be included in our approved ULT list: allopurinol, febuxostat, topiroxostat, benzbromarone, dotinurad, lesinurad, probenecid, sulfinpyrazone, pegloticase, and rasburicase.

**TABLE 1:** Urate-lowering therapeutic drugs approved in any country.

Medication	MOA	Approval	Company	Benefits	Drawbacks
Allopurinol	X	FDA, EMA, PMDA	Generic	Inexpensive, well-studied	Severe cutaneous allergic reactions
Febuxostat	X	FDA, EMA, PMDA	Takeda	Superior SUA-lowering effect relative to allopurinol	Black-box warning for increased risk of cardiovascular death
Topiroxostat	X	PMDA	Sanwa Kagaku Kenkyusho, Fuji Yakuhin	Novel alternative to allopurinol	Limited availability, twice daily dosing
Benzbromarone	U	EMA, PMDA	Sanofi-Aventis	Comparable SUA effect as allopurinol, superior to probenecid	Hepatotoxicity, limited worldwide availability
Lesinurad	U	FDA	AstraZeneca	Selective activity on URAT1	Not approved for monotherapy, removed from US market
Probenecid	U	FDA, EMA, PDMA	Lannett	Well-tolerated, long history of use	Nephrolithiasis, drug-drug interactions
Sulfinpyrazone	U	FDA, EMA	Novartis	–	Acute kidney injury, removed from US market
Pegloticase	R	FDA	Horizon Therapeutics	No need to renally adjust dose	Infusion reactions, ADA, high cost

MOA, mechanism of action; X, xanthine oxidase inhibitor; U, uricosuric; R, recombinant uricase; FDA, US Food and Drug Administration; EMA, european medicine administration; SUA, serum uric acid; ADA, antidrug antibodies.

We generated a list of ULT medications in development by searching ClinicalTrials.gov using the keywords “hyperuricemia” or “gout.” We excluded agents with purely anti-inflammatory activity. This search yielded 26 therapeutics: 17 had active or recently completed trials, and 9 were in the preclinical phase of development. After additional search for each drug name on PubMed and EMBASE and supplement data provided by companies, data related to the total of 36 medications was compiled.

## Results

### Approved Xanthine Oxidoreductase Inhibitors

#### Allopurinol

**FIGURE 1:**
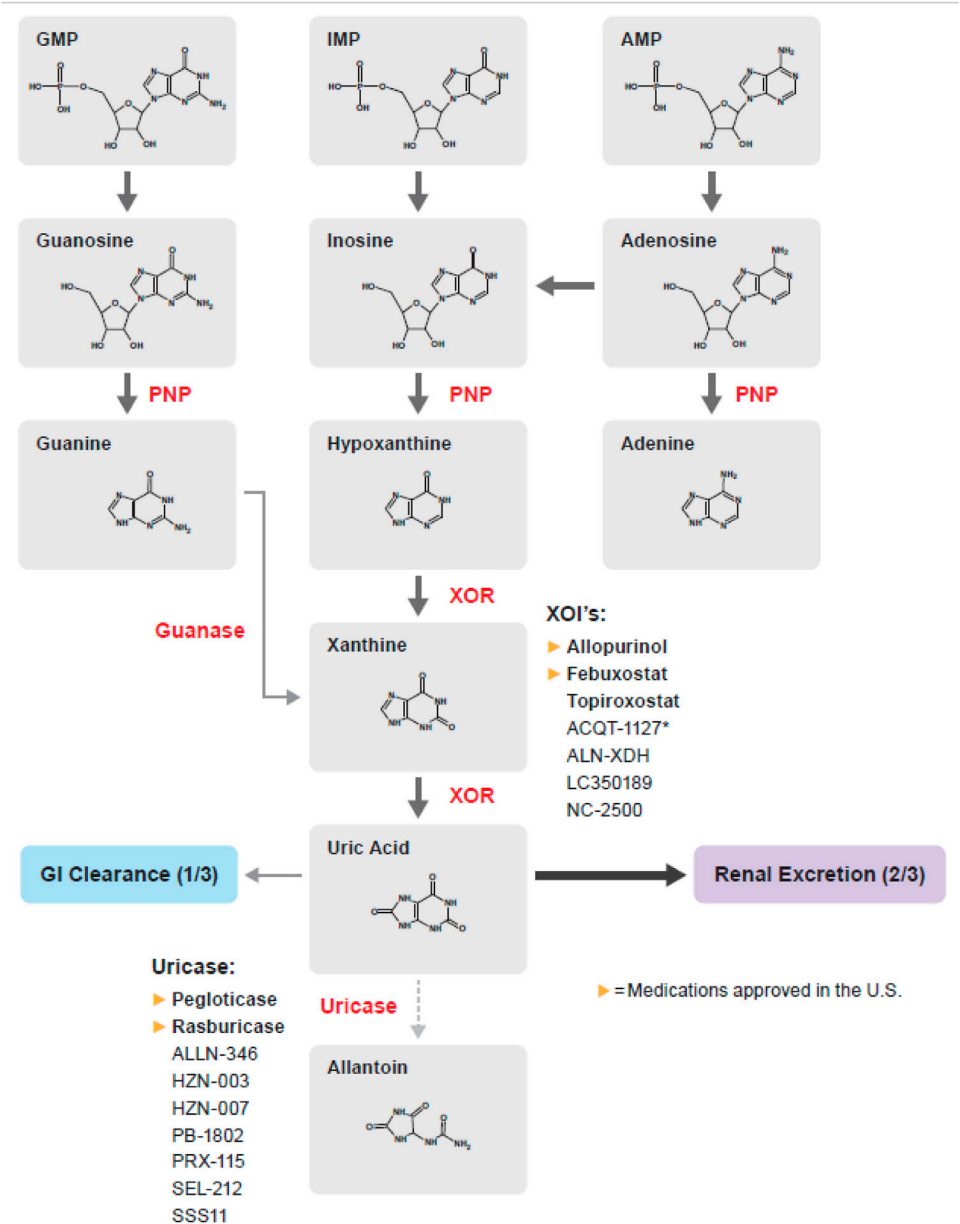
Purine metabolism pathway; medications in bold are approved by the FDA, EMA, or PDMA. AMP, Adenosine monophosphate; GMP, Guanosine monophosphate; IMP, Inosine monophosphate; PNP, Purine nucleoside phosphorylase; XOR, Xanthine oxidoreductase.

Allopurinol is a competitive inhibitor of xanthine oxidoreductase (XOR) (Figure 1). The 2020 American College of Rheumatology (ACR) guidelines recommend a starting dose of 100 mg daily, and titrating every 2–4 weeks until target SUA concentration is achieved with maximum daily dose of 800 mg (FitzGerald et al., 2020).

Primary side effects are gastrointestinal upset and skin rash. Allopurinol can cause severe cutaneous adverse reactions (SCAR), including drug rash with eosinophilia and systemic symptoms, Stevens-Johnson syndrome, toxic endodermal necrolysis, and allopurinol hypersensitivity syndrome. Mild skin rash and SCAR occur in approximately 2 and 0.4% of patients, respectively (Ramasamy et al., 2013). The incidence of severe reactions is highest during the first 2 months of therapy, and is associated with advanced age, renal dysfunction, and presence of the HLA-B*5,801 variant (Roujeau et al., 1995; Strilchuk et al., 2019; Stamp and Chapman, 2020). The 2020 ACR guidelines conditionally recommend testing for the HLA-B*5,801 allele prior initiating allopurinol in patients of Han Chinese, Korean, Thai, and African descent, as these populations have the highest prevalence (FitzGerald et al., 2020).

Allopurinol may slow the progression of CKD as seen in early data (Goicoechea et al., 2010). Two recent trials, Preventing Early Renal Loss in Diabetes (PERL) and A randomized Controlled trial of slowing of Kidney Disease progression From the Inhibition of Xanthine oxidase (CKD-FIX), did not find evidence that allopurinol slowed CKD progression (Badve et al., 2020; Doria et al., 2020), thus the benefit of allopurinol for renal function is currently unclear. Pooled analysis from these two trials raised concern that allopurinol possibly increased the mortality in patients with CKD. However, two large cohort studies concluded that allopurinol unlikely deteriorates renal function or increases mortality in patients with CKD (Vargas-Santos et al., 2018; Wei et al., 2022). Allopurinol initiation is not associated with neither preventing nor increasing CKD related outcomes.

Trials assessing allopurinol’s effects on cardiovascular disease have yielded mixed results. Studies have shown that treatment with allopurinol significantly improved brachial artery flow-mediated dilation (Cicero et al., 2018), increased exercise tolerance in subjects with chronic stable angina and coronary artery disease (Noman et al., 2010), reduced the rates of stroke and cardiac events in older adults with hypertension (MacIsaac et al., 2016), and improved survival in subjects with heart failure (Gotsman et al., 2012). By contrast, two studies did not demonstrate improvement in a clinical composite of cardiovascular outcomes in subjects with systolic, symptomatic heart failure (Hare et al., 2008; Givertz et al., 2015). The ALL-HEART trial is currently underway to determine allopurinol’s effect in subjects with ischemic heart disease (Mackenzie et al., 2016). Allopurinol remains the primary ULT due to its long track record of safety and efficacy, with the potential to improve outcomes in patients with cardiovascular disease.

#### Febuxostat

Febuxostat is a nonpurine, noncompetitive inhibitor of XOR (Frampton, 2015). The 2020 ACR guidelines recommend a starting dose of 40 mg daily and titrating until target SUA is achieved with typical daily doses of 80–120 mg (FitzGerald et al., 2020).

Several trials comparing febuxostat to allopurinol demonstrated that febuxostat was superior for SUA-lowering activity and tolerability, but was associated with increased adverse cardiovascular outcomes (Becker et al., 2005; Schumacher et al., 2008; Schumacher et al., 2009). The Cardiovascular Safety of Febuxostat and Allopurinol in Patients with Gout and Cardiovascular Morbidities (CARES) trial showed that febuxostat was noninferior to allopurinol for the primary composite outcome of cardiovascular events; however, all-cause and cardiovascular mortality rates were higher in the febuxostat group (White et al., 2018). The CARES trial outcomes are difficult to generalize due to the high discontinuation rate, loss to follow-up, lack of a placebo group, and suboptimal prescription rates of cardioprotective medications such as aspirin and beta blockers (Katsiki and Borghi, 2018; Abeles and Pillinger, 2019). A cohort study of 99,744 patients showed that febuxostat and allopurinol had statistically similar risks of myocardial infarction, stroke, new-onset heart failure, need for coronary revascularization, and all-cause mortality, but those taking febuxostat for more than 3 years had higher though statistically insignificant risk of all-cause mortality (Zhang et al., 2018). Based on these studies, the FDA placed a black-box warning on febuxostat for increased risk of cardiovascular death.

Febuxostat for Cerebral and CaRdiorenovascular Events PrEvEntion StuDy (FREED) and Febuxostat versus Allopurinol Streamlined Trial (FAST) demonstrated that febuxostat was noninferior to allopurinol with respect to a composite of cardiovascular outcomes, including cardiovascular death (Kojima et al., 2019; Mackenzie et al., 2020). The primary difference between the FAST and CARES trial is that all patients in the CARES trial had pre-existing CVD, while only 33.4% of participants in the FAST trial had CVD. FREED trial recruited eldery patients with hyperuricemia but not gout. Recent real-world data cohort trial showed that febuxostat did not increase CVD risk compared with allopurinol (Pawar et al., 2021). Further study is warranted to investigate this discrepancy.

Other studies reported that febuxostat improved morning home blood pressure, had renal protective effects in CKD, and inhibited the NLRP3 inflammasome *in vitro* (Lin et al., 2019; Nomura et al., 2019; Hsu et al., 2020; Kario et al., 2021). Additionally, both febuxostat and allopurinol maintained stable carotid-femoral pulse wave velocity over a 36-weeks treatment period (Desideri et al., 2022). Despite its superior urate-lowering effect and adverse effect profile, the higher cost and black-box warning make febuxostat an alternative to allopurinol according to the ACR 2020 guideline (Singh et al., 2015; FitzGerald et al., 2020).

#### Topiroxostat

Topiroxostat, a noncompetitive inhibitor of XOR (Okamoto et al., 2004), is currently approved for management of gout and hyperuricemia only in Japan. The maintenance dose is 60 mg twice daily, with a maximum dose of 80 mg twice daily. Several phase 2 trials showed that topiroxostat had a dose-dependent SUA lowering effect with similar safety profiles to allopurinol and placebo (Hosoya et al., 2016a; Hosoya et al., 2017). A phase 3 study demonstrated that 120 mg of topiroxostat daily was noninferior to 200 mg of allopurinol daily with respect to the SUA lowering effect (Hosoya et al., 2016b). The Cross-Over Trial of Febuxostat and Topiroxostat for Hyperuricemia with Cardiovascular Disease (TROFEO) trial showed that subjects taking febuxostat achieved more rapid reduction in SUA and required fewer dose adjustments compare to topiroxostat (Sezai et al., 2017) through greater XOR inhibition.

Topiroxostat has shown benefit in cardiovascular and renal outcomes. A study demonstrated a statistically significant increase in brachial artery flow-mediated dilation after 8 weeks of therapy (Higa et al., 2019). A trial of 123 subjects with stage 3 CKD and the Effect of Topiroxostat on Urinary albumin in hyperuricemic patients with Diabetic nEphropathy (ETUDE) trial found topiroxostat reduced urinary albumin-creatinine ratio (UACR) (Hosoya et al., 2014; Mizukoshi et al., 2018). The UPWARD trial did not show that topiroxostat reduced UACR, however it did slow the decrease in eGFR in patients with hyperuricemia and diabetic nephropathy with microalbuminuria (Wada et al., 2018).

The Beneficial Effect by Xanthine Oxidase Inhibitor on Endothelial Function Beyond Uric Acid (BEYOND-UA) study compared topiroxostat and febuxostat with respect to multiple cardiovascular and renal outcomes. Topiroxostat led to a superior decrease of morning home blood pressure and UACR compared to febuxostat (Kario et al., 2021). Topiroxostat has a safety profile similar to allopurinol and febuxostat, and potentially offers cardiovascular and renal benefits. In the future, we expect that topiroxostat could be a viable alternative to allopurinol and febuxostat.

### Approved Uricosuric Agents

#### Benzbromarone

**FIGURE 2:**
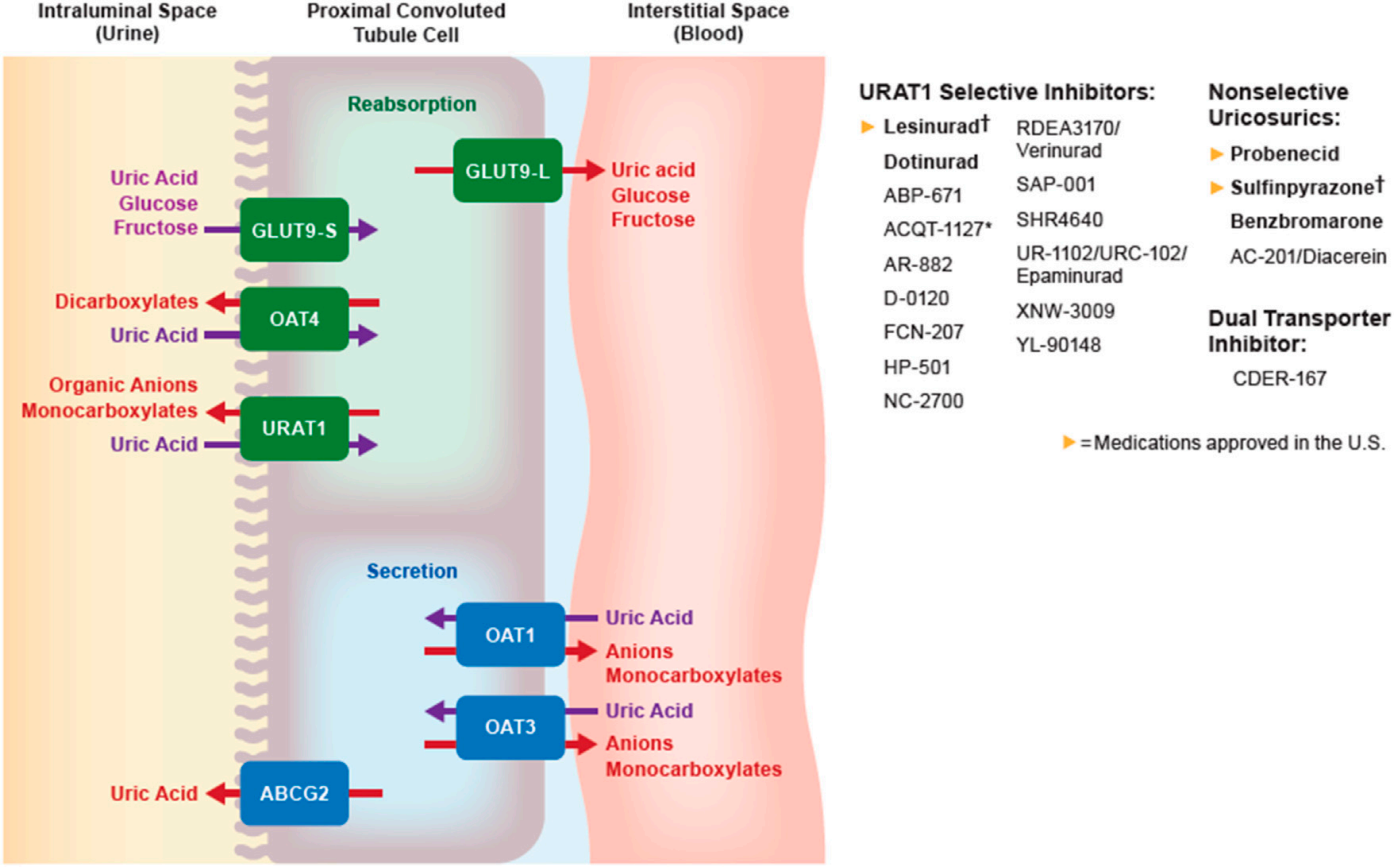
Urate transportasome in the proximal convoluted tubule; medications in bold are approved by the FDA, EMA, or PDMA. † denotes medications that are approved but no longer manufactured. ABCG, ATP binding cassette subfamily G; GLUT, Glucose transporter; OAT, Organic anion transporter (L, long; S, short); URAT, Urate transporter.

Benzbromarone is a nonselective uricosuric that lowers SUA by inhibiting *URAT1*, with a lesser effect on *GLUT9*, *OAT1*, and *OAT3* (Figure 2). The starting dose is 50 mg daily, with a maximum dose of 200 mg daily. Benzbromarone was never approved in the US due to potential hepatotoxicity, although it has been approved in Asia and Europe. The incidence of severe hepatoxicity has been estimated at 1 in 17,000 (Lee et al., 2008). The urate-lowering effect of benzbromarone is comparable to that of titrated doses of allopurinol, and superior to that of probenecid (Perez-Ruiz et al., 1998; Reinders et al., 2009; Kydd et al., 2014).

A small double-blind, placebo-controlled trial of subjects with heart failure with reduced ejection fraction treated with benzbromarone did not demonstrate any improvement in brain natriuretic peptide, left ventricular ejection fraction, echocardiographic assessment of cardiac dimensions, despite a significant decrease in SUA (Ogino et al., 2010). Based on its comparable efficacy to allopurinol and the relatively low rate of hepatotoxicity, benzbromarone could be considered for licensing in the US after additional safety studies are completed.

#### Probenecid

Probenecid is a nonspecific uricosuric agent that lowers urate primarily by inhibiting *URAT1* and other anion transporters (*OAT1*, *OAT3*, and *GLUT9*). Probenecid can be used as monotherapy if patients cannot tolerate XOIs or do not reach target SUA after XOI monotherapy (FitzGerald et al., 2020). Probenecid should be started at 500 mg once or twice daily, and subsequently titrated to a maximum dose of 2 g per day to reach target SUA. Stage 3 CKD or greater is a relative contraindication to probenecid use, although a small study showed no difference in SUA-lowering activity between subjects with an eGFR less than 50 ml/min/1.73 m^2^ compared with those with eGFR greater than 50 ml/min/1.73 m^2^ (Pui et al., 2013). Probenecid has been well-tolerated since it was introduced (Boger and Strickland, 1955; Pui et al., 2013). Two important adverse effects are urolithiasis and drug-drug interactions. Probenecid interacts with multiple transporters and alters the clearance of other medications, including penicillin, furosemide, and methotrexate (Homeida et al., 1977; Aherne et al., 1978; Overbosch et al., 1988).

Interim analysis of the Re-Prosper HF trial showed that probenecid led to a statistically significant improvement in systolic function, particularly in subjects with severely reduced ejection fraction (less than 25%) (Gilbert et al., 2015). Probenecid is a second-line agent in the management of gout due to the risk of urolithiasis, drug-drug interactions, and lower potency than other ULTs.

#### Lesinurad

Lesinurad was the first selective urate reabsorption inhibitor (SURI) (Miner et al., 2016), and was typically administered as a 200 mg daily dose in combination with a XOI. Lesinurad does not alter the function of OAT1 or OAT3 transporters, unlike probenecid (Yeh, 2009; Miner et al., 2016). It gained FDA approval in 2015 for use in combination with allopurinol or febuxostat, although production was discontinued in 2019 by the manufacturer. The FDA database states that this discontinuation was not related to drug safety or efficacy.

#### Sulfinpyrazone

Sulfinpyrazone lowers SUA by inhibiting URAT1. The starting dose was 50 mg twice daily, with a total maximum daily dose of 800 mg. Like lesinurad, sulfinpyrazone was discontinued by the manufacturer without documented adverse safety or efficacy events, although several cases of acute renal failure were attributed to sulfinpyrazone (Prior and Kirchmair, 1984; Walls et al., 1998).

#### Dotinurad (FYU-981)

Dotinurad is a SURI (Fuji Yakuhin, Chiba, Japan) that was approved in Japan 2020 (Taniguchi et al., 2019). *In vitro* studies reported that dotinurad also inhibits the NLRP3 inflammasome (Taufiq et al., 2020). Two phase 2 studies demonstrated dose-dependent urate-lowering effects in subjects taking 0.5–4 mg/d of dotinurad (Kuriyama, 2020). Two phase 3 trials showed that dotinurad was noninferior to febuxostat or benzbromarone in SUA-lowering activity and adverse effects (Hosoya et al., 2020a; Hosoya et al., 2020b). These studies also found that renal function did not affect SUA-lowering effect. Dotinurad was recently licensed (Fortress Biotech, New York, NY) for additional development in North America.

### Approved Recombinant Uricases

#### Rasburicase

Rasburicase is the prototypical recombinant uricase. It is approved for the treatment of hyperuricemia in malignancy and tumor lysis syndrome. The typical dose is 2.0 mg/kg intravenous daily for one to 5 days based on clinical symptoms. Rasburicase is usually well-tolerated, although it does have a black-box warning for anaphylaxis, hemolysis, and methemoglobinemia (Inc, 2009). Rasburicase has a potent and rapid urate-lowering effect superior to allopurinol, particularly within 4 hours of administration (Goldman et al., 2001; Cortes et al., 2010). As with most other recombinant therapeutics, rasburicase is immunogenic which can lead to the development of anti-rasburicase antibodies in 11–64% of patients (Pui et al., 2001; Inc, 2009; Allen et al., 2015). Repeat courses of rasburicase are not recommended due to the increased rate of anaphylaxis in patients receiving a subsequent course (Allen et al., 2015).

A small exploratory study of participants with severe tophaceous gout and CKD received monthly infusions of 2.0 mg/kg rasburicase. The results showed a trend toward achieving SUA of 6.0 mg/dl and decreased tophus size (Richette et al., 2007). No additional similar trials have been completed since repeat courses are not recommended. Rasburicase is not a preferred agent for long-term management of gout due to limited therapeutic indication, cost, and route of administration.

#### Pegloticase

Pegloticase is a recombinant uricase conjugated with polyethylene glycol (PEG) approved for use in severe gout refractory to oral ULT. The 2020 ACR guidelines strongly recommend pegloticase for patients who continue to have frequent flares or nonresolving tophi despite compliance with maximally tolerated XOI, uricosurics, combination therapy, and other interventions (FitzGerald et al., 2020). Unlike most other ULT, pegloticase does not require dose adjustment based on renal function, and has a substantially longer half-life than rasburicase. The typical dose is 8 mg intravenously every 2 weeks. Two phase 3 trials compared biweekly infusions and monthly infusions of pegloticase with placebo. The twice-monthly infusion protocol was superior to placebo in achieving SUA less than 6 mg/dl for at least 80% of the time between months 3 and 6 of the study, as well as the secondary outcomes of tophus resolution and flare incidence (Sundy et al., 2011).

Some limitations in pegloticase therapy include 2-h infusion duration, twice-monthly infusions, high cost, relatively frequent infusion reactions including anaphylaxis, incompatibility in patients with glucose-6-phosphate dehydrogenase deficiency, development of antidrug antibodies (ADA), and loss of urate-lowering efficacy (Lyseng-Williamson, 2011). The most common infusion reactions are chest discomfort, flushing, and dyspnea, which typically resolve with slowing, pausing, or discontinuing infusion (Baraf et al., 2014). Development of ADA is related to incidence of infusion reactions and tachyphylaxis (Baraf et al., 2014; Lipsky et al., 2014). This has prompted “stopping rules,” whereby pegloticase therapy is discontinued if pre-infusion SUA is greater than 6 mg/dL. A prospective study reported only one infusion reaction in 315 infusions when following this guidance (Saag et al., 2017).

There are several ongoing efforts to reduce the immunogenicity of pegloticase. The Methotrexate to Increase Response Rates in Patients With Uncontrolled Gout Receiving KRYSTEXXA Open Label (MIRROR OL) trial showed that 11/14 (78.6%) of subjects achieved a SUA less than 6 mg/dl for at least 80% of the time during month 6, compared to previously reported 42% of patients on pegloticase alone (Botson et al., 2020). This led to the MIRROR-RCT (NCT03994731), Tolerization Reduces Intolerance to Pegloticase and Prolongs the Urate Lowering Effect (TRIPLE) trial using azathioprine (NCT02598596), and the REduCing Immunogenicity to PegloticasE (RECIPE) trial with mycophenolate mofetil (NCT03303989). Patients receiving pegloticase experience statistically significant and clinically meaningful improvement in several metrics of disease burden and symptomatology related to chronic gout (Strand et al., 2012). Therefore, the use of pegloticase in clinical practice should continue despite associated concerns.

### Medications Currently Undergoing Development

Drugs in clinical development stage are listed in Table 2 and drugs in preclinical development stage are listed in Table 3. Agents that have published data are included in the body of the review, while those without are only mentioned in the tables.

**TABLE 2:** Developmental pipeline for urate-lowering therapeutic agents in the clinical phase of development.

Compound	Name	Company	MOA	Phase	Highest phase NCTand status	NCTLast update	Website
LC350189	—	LG Chem	X	2	NCT03934099, Completed	13 Jan 2022	innovation.lgchem.com
NC-2500	—	Nippon Chemiphar	X	1	–	–	chemiphar.co.jp
ABP-671	—	Jiangsu Atom Bioscience and Pharmaceutical	U	2	NCT04638543, Recruiting	20 Nov 2020	www.atombp.com
AC201	Diacerein	TWi Pharmaceuticals	U, A	2	NCT02287818, Completed	28 Oct 2020	twipharma.com
AR-882	—	Arthrosi Therapeutics	U	2	NCT05119686, Recruiting	17 Feb 2022	arthrosis.com
D-0120	—	InventisBio	U	2	NCT03923868, Recruiting	Sept 29, 2020	inventisbio.com
FCN-207	—	Fochon Pharmaceuticals	U	1	NCT04622124, Recruiting	21 Dec 2021	fochon.com
HP-501	—	Hinova Pharmaceuticals	U	2	China Phase 2	–	hinovapharma.com
RDEA3170	Verinurad	*Ardea*, AstraZeneca	U	2	NCT03118739, Completed	10 Jan 2020	astrazeneca.com
SAP-001	—	Shanton Pharma	U, A	2	NCT04040816, Completed	5 Nov 2021	shantonpharma.com
SHR4640	—	Jiangsu HengRui Medicine	U	3	NCT04956432, Recruiting	9 Jul 2021	hengruitx.com
UR-1102/URC-102/SIM-295	Epaminurad	JW Pharmaceutical	U	2	NCT04804111, Completed	18 Mar 2021	jw-pharma.co.kr
XNW-3009	—	Sinovent	U	1	NCT04040907, Complete	6 Jan 2022	linear.org.au
YL-90148	—	Shanghai YingLi Pharmaceutical	U	2	–	–	yl-pharma.com
ALLN-346	—	Allena Pharmaceuticals	R	2	NCT04987294, Recruiting	6 Dec 2021	allenapharma.com
SEL-212	Pegadricase + ImmTOR™	Selecta Biosciences	R	3	NCT04596540 and NCT04513366, Recruiting	5 April 2022	selectabio.com

MOA, mechanism of action; X, xanthine oxidase inhibitor; U, uricosuric agent; A, anti-inflammatory; R, recombinant uricase.

**TABLE 3:** Developmental pipeline for urate-lowering therapeutic agents in the clinical phase of development.

Compound	Company	MOA	Website
ALN-XDH	Alnylam Pharmaceuticals	X	alnylam.com
CDER167	—	U	—
NC-2700	Nippon Chemiphar	U	chemiphar.co.jp
HZN-003/MEDI-4945	Horizon Therapeutics	R	horizontherapeutics.com
HZN-007/XL-400	Horizon Therapeutics	R	horizontherapeutics.com
PB-1802	Chongqing Peg-Bio Biotech	R	pegbio.com
PRX-115	Protalix Biotherapeutics	R	protalixbiotherapeutics.gcs-web.com
ACQT-1127/RLBN-1127	Acquist Therapeutics	U, X	acquistrx.com

MOA, mechanism of action; X, xanthine oxidase inhibitor; U, uricosuric agent; R, recombinant uricase.

#### Novel XOIs

##### LC350189

LC350189 is an XOI (LG Life Science, Seoul, South Korea). A phase 1 study demonstrated tolerability at a wide range of doses (10–800 mg) and efficacy in lowering SUA (Yoon et al., 2015). Four additional phase 1 trials (NCT03927677, NCT04139824, NCT04070846, NCT04066712), and a phase 2 efficacy and safety trial (NCT03934099) have been completed.

##### NC-2500

NC-2500 is an XOI (Nippon Chemiphar, Tokyo, Japan). A phase 1 trial showed a near dose-dependent urate-lowering effect over a range of 10–160 mg with side effects similar to placebo (Hirano et al., 2018a).

##### TMX-049

TMX-049 is an XOI (Teijin Pharma, Tokyo, Japan). A phase 1 single dose trial showed a dose dependent decrease in SUA in doses ranging from 10 to 380 mg (Center, 2018). A phase 2 study reported that 200 mg of TMX-049 daily led to a statistically significant reduction in UACR after 12 weeks compared to placebo (Bakris et al., 2020a).

#### Novel Uricosurics

##### ABP-671

ABP-671 is a URAT1 inhibitor (Atom Bioscience, Jiangsu, China). Three phase 1 trials have been completed (NCT04303039, NCT04060173, NCT03906006) with dosages ranging from 0.1 to 50 mg. A phase 2 clinical trial is currently recruiting participants (NCT04638543).

##### AR-882

AR-882 is a URAT1 inhibitor (Arthrosi Therapeutics, San Diego, CA). Initial phase 1 data reported statistically significant decrease in SUA at 24 h after administration of doses greater than 50 mg, with only mild adverse effects (Shen et al., 2019). A phase 1, multiple ascending dose trial demonstrated significant SUA-lowering effect over a 10-days treatment period at doses of 25 mg, 50 mg, and 75 mg daily when compared to placebo (Shen et al., 2020). Two other phase 1 trials (NCT04347005 and NCT04508426) and a phase 2 trial (NCT04155918) are complete. An additional phase 1 trial assessing its pharmacokinetics in renal impairment is currently recruiting (NCT04646889).

##### CDER167

CDER167 is a dual-acting uricosuric targeting URAT1 and GLUT9, which is in the preclinical phase of development. In vivo experiments in rats demonstrated SUA-lowering activity and relative safety (Zhao et al., 2021).

##### D-0120

D-0120 is a novel selective URAT1 inhibitor in phase 2 of development (InventisBio, Shanghai, China). A phase 1 trial showed it was well-tolerated and a phase 2 clinical trial is currently recruiting participants (NCT03923868) (Zhang et al., 2020).

##### NC-2700

NC-2700 is a SURI (Nippon Chemiphar, Tokyo, Japan). *In vivo* studies showed a dose-dependent increase in urinary uric acid excretion in tufted capuchin monkeys, and increased urinary pH in rats (Hirano et al., 2018b). Urine alkalinization increases the solubility of uric acid, reducing the risk of urolithiasis.

##### RDEA3170 (Verinurad)

Verinurad is a SURI (AstraZeneca, Cambridge, United Kingdom). Verinurad is three times more potent than benzbromarone and 100 times more potent than probenecid (Miner and Tan, 2013). Phase 1 trial demonstrated sustained urate-lowering activity and tolerability at doses ranging from 2.5 to 15 mg (Shen et al., 2017; Hall et al., 2018). Several trials assessing verinurad in combination with allopurinol or febuxostat showed superior urate-lowering effects relative to monotherapy (Fleischmann et al., 2018; Kankam et al., 2018; Shiramoto et al., 2018; Fitz-Patrick et al., 2019; Hall et al., 2019). Verinurad plus febuxostat reduced albuminuria and lowered SUA in subjects with type 2 diabetes mellitus, albuminuria, and hyperuricemia (Stack et al., 2021). The Study of Verinurad in Heart Failure with Preserved Ejection Fraction (AMETHYST) trial is currently recruiting to assess the effect of verinurad plus allopurinol on exercise capacity in subjects with heart failure with preserved ejection fraction (NCT04327024).

##### SHR4640

SHR4640 is a URAT1 inhibitor (Jiangsu Hengrui Medicine Company, Jiangsu, China). Eight phase 1 studies have been completed (NCT04260373 (Miner and Tan, 2013)), NCT03015948, NCT02815839, NCT03211403, NCT02890966, NCT03131583, NCT04157959, and NCT04305392. A phase 2 trial (NCT03185793) reported that SHR4640 at doses of 5 and 10 mg achieved target SUA (less than 6 mg/dl) compared to placebo (Lin et al., 2021). A phase 3 trial (NCT04052932) is currently recruiting.

##### UR-1102/URC-102 (Epaminurad)

Epaminurad is a SURI (JW Pharmaceuticals, Seoul, South Korea). *In vitro* and animal studies demonstrated superiority in lowering SUA and increasing fractional excretion of uric acid compared to benzbromarone (Ahn et al., 2013; Ahn et al., 2016). Epaminurad doses ranging from 1 to 10 mg were well-tolerated and achieved sustained dose-dependent urate-lowering effects (Lee et al., 2019). Three phase 2 trials assessing safety, pharmacokinetics, and pharmacodynamics have been completed (NCT02290210, NCT02557126, and NCT04804111). A phase 1 trial assessing safety in individuals with renal impairment is currently recruiting (NCT05198778). Phase 3 is under preparation.

##### XNW-3009

XNW-3009 is a URAT1 inhibitor (Sinovent, Jiangsu, China). A phase 1 study of XNW-3009 doses ranging from 1 to 50 mg has been completed (NCT04040907), and an additional phase 1 trial assessing drug-drug interactions with febuxostat and colchicine is recruiting (NCT05324423).

#### Novel Recombinant Uricases

##### ALLN-346

ALLN-346 is an orally administered recombinant uricase (Allena Pharmaceuticals, Newton, MA). This is based on recognition that the gastrointestinal tract is a major source of SUA excretion in patients with CKD (Pierzynowska et al., 2020). Two phase 1 trials have been completed (NCT04829435 and NCT04236219) and two phase 2 trials are recruiting participants (NCT04987294 and NCT04987242).

##### SEL-212 (Pegadricase + ImmTOR)

SEL-212 is a combination therapy of a PEGylated recombinant uricase and an immune tolerance platform (synthetic vaccine particle encapsulating rapamycin, SVP-R). (Selecta Biosciences, Watertown, MA). Phase 1 data showed dose-dependent inhibition of ADA and a sustained urate-lowering effect (Sands et al., 2017). The majority of subjects in a phase 2 study receiving monthly injections of SEL-212 maintained a SUA of less than 6.0 mg/dl at 20 weeks, with low rates of ADA and infrequent gout flares (Smolinski et al., 2019). Two phase 3 trials to determine the safety and efficacy of two different doses of SEL-212 are recruiting (DISSOLVE I (NCT04513366) and DISSOLVE II (NCT04596540)).

##### HZN-003 (MEDI-4945)

HZN-003 is a recombinant uricase in the preclinical phase of development (Horizon Therapeutics, Dublin, Ireland). According to the company’s website, this is an upgraded version of pegloticase that uses a genetically engineered uricase and optimized PEGylation technology with the potential to improve half-life and reduce immunogenicity.

##### HZN-007

HZN-007 is a PASylated recombinant uricase in the preclinical phase of development (joint effort by Horizon Therapeutics, Dublin, Ireland, and XL-Protein, Freising, Germany). PASylation is a biological alternative to PEGylation, which utilizes a repeating proline-alanine-serine (PAS) motif to prolong half-life and combat immunogenicity that is inherent to PEGylation (Schlapschy et al., 2013).

##### PRX-115

PRX-115 is a PEGylated recombinant uricase in the preclinical phase of development (Protalix Biotherapeutics, Karmiel, Israel). Preclinical data suggested prolonged half-life, lower ADA, and effective SUA-lowering activity (Biotherapeutics, 2021).

#### Novel Agents With Multiple Mechanisms

##### AC-201 (Diacerein)

Diacerein is a uricosuric and an anti-inflammatory (TWi Pharmaceuticals, Taipei, Taiwan). It has inhibitory effects on URAT1, caspase 1, and IL-1β. Diacerein has been available for several years in Europe and Asia for “background treatment” of osteoarthritis at a dose of 50 mg once or twice daily. A meta-analysis showed that diacerein had similar efficacy as nonsteroidal anti-inflammatory drugs for management of knee and hip osteoarthritis (Pavelka et al., 2016) although its use is restricted due to the occurrence of severe diarrhea and liver dysfunction. Data from a phase 2 clinical trial showed that febuxostat plus 100 mg of diacerein twice daily had better achievement of target SUA than febuxostat plus placebo.

##### CDER167

CDER167 is a dual-acting uricosuric targeting URAT1 and GLUT9, which is in the preclinical phase of development. *In vivo* experiments in rats demonstrated SUA-lowering activity and relative safety (Zhao et al., 2021).

## Discussion

ULTs are the mainstay of chronic gout management because they directly target the disease etiology. The high failure rate of the treat-to-target approach has been attributed to patient and provider knowledge gaps, pre-existing severely elevated SUA, concomitant medical conditions such as CKD, and precipitation of disease flares. Women and patients of minority ethnicities are underrepresented in ULT trials, which affects the generalizability of these studies (Fogacci et al., 2021).

Many currently available ULT have large clinical trials demonstrating their efficacy and tolerability, particularly in patients with advanced age, several comorbidities, and multiple medications (Cicero et al., 2021a). Each of these agents has differences in efficacy, tolerability, and side effect profiles (Cicero et al., 2021b). These concerns necessitate a new wave of ULT.

There have been several changes to the ULT pipeline during the past 5 years. The parent companies developing arhalofenate and ulodesine recently halted research despite completing multiple phase 2 trials. The release of several new biologic agents for other rheumatic diseases has generated interest in developing injectable ULTs, such as subcutaneous uricase.

Developing more potent, better tolerated, and dual-functioning ULT with anti-inflammatory effects (e.g., AC-201 and SAP-001) will be a key strategy for next-generation therapeutics as it will improve adherence to treatment, increase the likelihood of achieving target SUA, and reduce the frequency of disease flares. Continuing research on the renal and intestinal urate transportasomes will provide future therapeutic targets, such as SMCT1/2 (Lu et al., 2013), ABCG2 (Nakayama et al., 2011; Matsuo et al., 2014; Bhatnagar et al., 2016), and GLUT-9 (Preitner et al., 2009).

ULTs have the potential to offer benefits beyond gout. This interest in cardiovascular and renal benefits likely stems from several recent high-profile trials involving sodium-glucose transport protein 2 (SGLT2) inhibitors and novel selective mineralocorticoid receptor antagonists (Wanner et al., 2016; Perkovic et al., 2019; Bakris et al., 2020b; Heerspink et al., 2020). Allopurinol has shown some improvement in cardiovascular outcomes and renoprotection (Hare et al., 2008; Goicoechea et al., 2010; Noman et al., 2010; Gotsman et al., 2012; Givertz et al., 2015; MacIsaac et al., 2016; Cicero et al., 2018; Vargas-Santos et al., 2018; Badve et al., 2020; Doria et al., 2020). Newer studies suggest that febuxostat also has some renal protection in CKD (Lin et al., 2019; Hsu et al., 2020). Topiroxostat has shown some benefit related to decreasing UACR in microalbuminuria (Hosoya et al., 2014; Mizukoshi et al., 2018; Wada et al., 2018; Higa et al., 2019; Kario et al., 2021). Based on interim results from the Re-Prosper trial, probenecid confers a significant improvement in systolic function in participants with severely reduced ejection fraction (Gilbert et al., 2015). Verinurad combined with febuxostat significantly reduced albuminuria (Stack et al., 2021). Conversely, a systematic review and meta-analysis of 28 trials including allopurinol, febuxostat, topiroxostat, lesinurad, and pegloticase did not find any significant improvement in cardiovascular events, death, or kidney failure compared to placebo, though it did demonstrate these agents improved systolic and diastolic blood pressure, as well as an attenuation of decline in GFR (Chen et al., 2020).

There are several novel approaches to reduce the immunogenicity of recombinant uricase for patients with refractory gout. ALLN-346, an oral formulation of uricase, should prevent the development of ADA by avoiding the blood stream. HZN-007 is expected to avoid ADA because PAS is a biosynthetic alternative to PEG. Coadministration of SVP-R along with SEL-212 reduces ADA formation. The MIRROR-RCT, TRIPLE, and RECIPE trials are underway to test whether combining pegloticase with methotrexate, azathioprine, and mycophenolate mofetil, respectively, reduces immunogenicity.

One limitation of our review is that it excluded medications with purely anti-inflammatory activity. The only classes of medications approved for treating or preventing flares are NSAIDs, glucocorticoids, IL-1 receptor antagonists, and colchicine. Each of these classes of medications is again associated with restrictions. The 2020 ACR guidelines strongly recommend flare prophylaxis for three to 6 months when initiating ULT (FitzGerald et al., 2020). Several therapeutic agents are currently in development for flare prophylaxis, but a comprehensive review of their properties was beyond the scope of this study. We also elected to exclude agents that lower urate as a secondary mechanism, such as losartan and SGLT2 inhibitors.

This article provides an updated review of all currently available ULTs and 26 medications in various stages of development. This review is a comprehensive guide to the properties, indications, and limitations of ULTs and the myriad options of new medications.
